# Association between dental fracture and amalgam restoration: a case-control study

**DOI:** 10.1590/1678-7757-2024-0467

**Published:** 2025-04-18

**Authors:** Luana dos Santos SOUZA, Victor RUANO, Rafael Santos ROCHA, Eduardo VARANDA, Taciana Marco Ferraz CANEPPELE, Eduardo BRESCIANI

**Affiliations:** 1 Universidade Estadual Paulista Instituto de Ciência e Tecnologia Departamento de Odontologia Restauradora São José dos Campos São Paulo Brasil Universidade Estadual Paulista, Instituto de Ciência e Tecnologia, Departamento de Odontologia Restauradora, São José dos Campos, São Paulo, Brasil.; 2 Universidade Federal do Rio de Janeiro Departamento de Odontologia Restauradora Rio de Janeiro Rio de Janeiro Brasil Universidade Federal do Rio de Janeiro, Departamento de Odontologia Restauradora, Rio de Janeiro, Rio de Janeiro, Brasil.

**Keywords:** Tooth fractures, Dental amalgam, Dental care, Dental materials

## Abstract

**Objective:**

To evaluate the possible clinical association between dental fracture and the presence of amalgam restorations, including other restorative treatments in the control group. The potential association of fractures with dental wear facets and the restoration size was also assessed as a secondary objective.

**Methodology:**

Patients with fractured teeth restored with silver amalgam or not were included as the case group (n=25). The control group, with non-fractured teeth, was selected after considering the case group aspects, with twice as many patients (n=50) with posterior teeth sound or restored (amalgam, composite resin, or another restorative material). For both groups, the type of restorative material, extension of the restorations, remaining tooth structure, and the presence or absence of wear facets were analyzed. The teeth were impressed with alginate, and from the plaster models, the extent of fractures or restorations was measured by two calibrated examiners with a digital caliper at the cervico-occlusal and bucco-lingual directions. The data were subjected to the Chi-square test (5%) and odds ratio.

**Results:**

There was no statistical difference between the presence or absence of amalgam restorations regarding the risk of tooth fracture. Regarding fractures larger than 3.5mm, the chances of failure are 0.53 for amalgam restorations with no statistical differences (p=0.433), and, regarding the presence of wear facets, the odds ratio of failure is 1.357 for amalgam restorations (p=0.65).

**Conclusion:**

It can be deduced that, within the conditions of the study, no discernible association exists between dental fractures and the presence of silver amalgam restorations.

Clinical Trial Register: (ReBEC) UNT code U1111-1215-7255.

**Figure:**
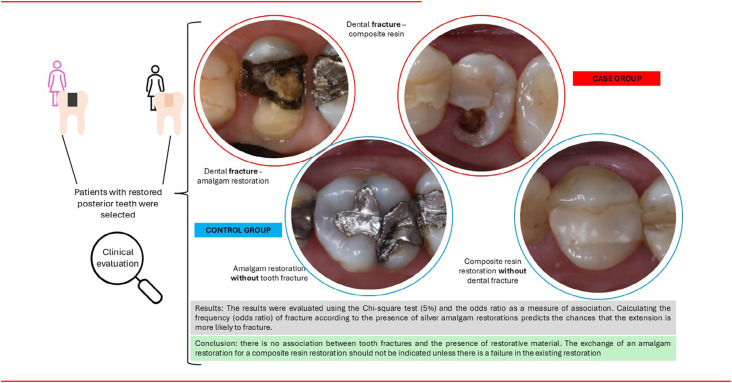


## Introduction

Dental crown fractures represent a significant clinical concern, characterized by the loss of dental structure. These fractures are classified as complete when the fractured portion detaches entirely from the tooth, or incomplete (cracks) if the broken portion remains in place.^1,2^ Clinically, complete dental fractures are easily diagnosed, while incomplete fractures are more complex. The latter may be diagnosed as "cracked tooth syndrome," depending on the clinical signs and symptoms.^2^

The causes of complete dental fractures are mainly related to factors such as sudden biting of hard objects, dental caries, or previously restored teeth. However, the type of anatomy and inclination of the cusps, traumatic occlusal relationships, endodontic treatment, and parafunctional habits (bruxism, clenching), are also related to tooth fracture.^3-5^ The risk of fracture is more frequent in teeth that have large restorations as less than 10% occur in sound teeth.^5-7^

Furthermore, root canal-treated teeth are more likely to fracture since characteristics like the loss of structure and the arching roof of the pulp chamber, and the endodontic access cavity preparations considerably increase the cuspal deflection in the tooth. Those factors consequently increase the risk of cusp breakage during function; the transmission of stresses can lead to fractures/fissures of various extents, which are directly correlated to the time of occurrence of complete fracture.^4,5,8,9^

While the type of restorative material is often considered a primary factor in the risk of dental fractures, a multitude of other variables, such as the extent of tooth structure loss, occlusal forces, and restorative techniques, also play crucial roles. Restorative failures may stem from differences in the modulus of elasticity between materials, such as silver amalgam and composite resin. For instance, composite restorations are more prone to secondary caries and polymerization shrinkage, in contrast, amalgam restorations may lead to fractures due to the brittleness of the material and the need for extensive cavity preparations.^9^ Despite these differences, clinical evidence does not establish a direct association between the presence of restorative materials and the occurrence of dental fractures.^10-13^

Amalgam, with a modulus of elasticity significantly higher than dentin, has been critiqued for potentially increasing fracture susceptibility. Despite its durability, esthetic limitations and mercury-related environmental concerns have driven its gradual replacement by adhesive materials such as composite resins.^14^ Although more esthetic, these materials are more technique-sensitive and prone to microleakage and secondary caries.^16^

Research suggests that cavity size and preparation technique are key determinants of fracture risk.^5,10,11^ Teeth with extensive restorations exhibit a higher risk of fracture compared to sound teeth; larger cavities further exacerbate this risk.^8^ Preparation for amalgam restorations typically requires removing more tooth structure, potentially weakening it-.^16^ Conversely, composite resin techniques, while more conservative, may lack long-term durability.^17^

Given these complexities, questions remain about the precise role of restorative materials in dental fractures. Current evidence is insufficient to conclusively link the presence of silver amalgam or composite resin restorations to fracture risks, necessitating robust clinical investigations. Understanding these relationships could inform safer restorative protocols, optimizing treatment outcomes.^18^

Given these observations regarding the multifactorial nature of fractures, the lack of confirmation on the relationship between tooth fracture and the presence of restorative materials, mainly due to the lack of clinical studies reporting this condition, there is a need for clinical answers on this possible correlation. It would help define a safer restorative protocol according to the indication and possible contraindication of the mentioned restorative procedures, and the indication of restoration replacement, mainly amalgam restoration.

Therefore, this case-control study aimed to investigate the possible association of dental fractures with the presence or absence of silver amalgam restorations and other materials (in the control group), as well as factors that may be related to tooth fractures, such as the thickness of the fractured tooth remnant, the presence of wear veneers and marginal cracks. The null hypothesis formulated was that there is no association between fractured teeth and the presence of silver amalgam restorations.

## Methodology

**Figure 1:**
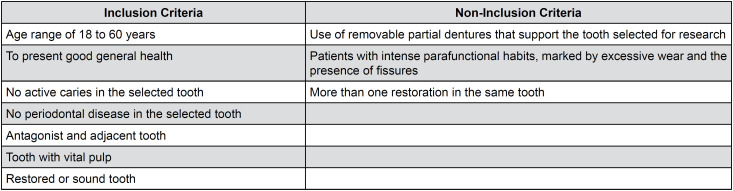
Inclusion and exclusion criteria.

The sample size was calculated on the Lee—Laboratory of Epidemiology and Statistics (lee.dante.br) website (http://www.openepi.com/SampleSize/SSCC.htm). A dichotomous response analysis (comparison of proportions) for case-control studies was adopted, using unpaired, two-tailed characteristics, with the control group having twice as many selected teeth as the case group, with 80% power and adopting a 5% significance level. Considering the proportion of exposed teeth between cases (the presence of fractured tooth with amalgam restoration) as 90% and the proportion of exposed teeth among controls (non-fractured teeth with amalgam restoration) as 60%, 25 teeth were allocated to the case group and 50 teeth to the control group. This study was approved by the local ethics committee of the Institute of Science and Technology of São José dos Campos—UNESP and received approval with the identification number 13271919.8.0000.0077. This observational case-control study was registered in the Brazilian Trials of Clinical Registries (ReBEC) U1111-1215-7255 database and approved by the Institutional Review Board, following the recommendations of the Strobe Statement, and the research was conducted from March to September 2019. The inclusion and exclusion criteria are detailed in Figure 1. Notably, restored teeth, with or without fractures, were also considered an inclusion criterion.

For the case group, 25 patients presenting with fractured teeth (case group/n=25) and meeting the inclusion criteria were evaluated and included in the study. After including the participants of the case group, a second evaluation was conducted to select patients for the control group (n=50), following matching characteristics concerning age, gender, and group of teeth (molar and premolar). Patients were further categorized into two groups: those with amalgam restorations and those with composite resin and/or other restorative material or even sound tooth.

**Figure 2:**
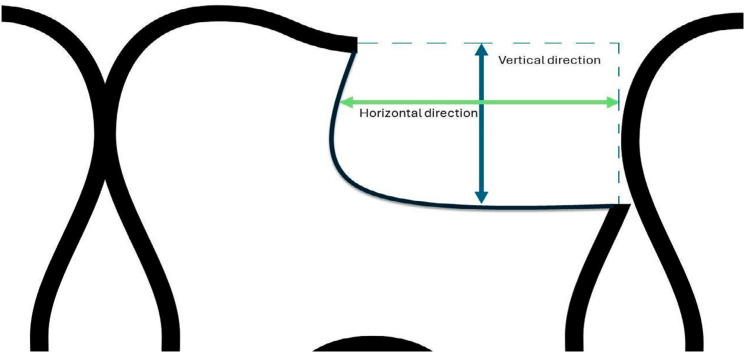
Schematic diagram simulating the measure of a fracture in the plaster model using a digital caliper. The blue arrow is the vertical direction, indicating the depth of the fracture and, the green arrow represents the width of the fracture.

To measure the cavity size resulting from fractures or remaining restorations (in the control group), all teeth received alginate impressions to create type IV plaster models. The extent of fractures was measured using a digital caliper in two orientations: horizontal (mesio-distal or buccal-lingual direction) and vertical (occlusal-cervical direction) (Figure 2). The caliper tips were positioned along the measurement axis on the plaster model, ensuring consistency by measuring from the most extensive region to the opposite end within the same direction, as illustrated in Figure 2. For restoration, the buccal-lingual size was determined by averaging three measurements taken at the mesial, central, and distal regions of the tooth. A professional camera (CANON T5, equipped with a 100mm macro lens and circular flash) was employed to document fractures when present.

For case group patient selection, teeth were clinically examined using a dental mirror and explorer in a clean, dry, and well-lit environment. Upon fracture confirmation, a detailed record was made, which included the presence of any restorative material. Without clinical evidence of restorative material, patient records were consulted to determine the material present before the fracture and to identify the fractured tooth surfaces. Subsequently, initial photographs were taken, followed by a full-arch alginate impression to fabricate type IV dental casts. On these casts, the vertical and horizontal extent of the fracture was measured using a digital caliper. Additionally, the presence of incisal wear on anterior teeth, previously observed clinically, was confirmed using the cast.

Following the selection of case group patients, control group patients were recruited. Posterior teeth without fractures, whether restored or intact, were included. The same clinical approach used for the case group was employed; regarding dental casts, the extent of restorations was measured using a digital caliper. Additionally, the restored tooth surfaces were recorded.

For secondary outcomes, the presence of wear facets characteristic of attrition on anterior teeth was clinically assessed in a clean, dry, and well-lit environment using a dental mirror and explorer. Attritional wear was classified as "present" or "absent," with "present" being defined as incisal wear affecting two or more anterior teeth and confirmed on dental casts. Dental casts provide easier visualization of dental facets due to better plaster contrast. The pilot study revealed that restorations larger than 3.5 mm in overall extension typically encompassed two or more surfaces, necessitating more extensive preparations and restorations. This reduction in the remaining tooth structure could predispose the tooth to fracture. No information in the literature supports the mentioned data, which is why that measure was decided on a pilot observation.

The results were evaluated using the Chi-square test (5%) and the odds ratio as a measure of association. Calculating the frequency (odds ratio) of fracture according to the presence of silver amalgam restorations predicts the chances that the extension is more likely to fracture. This calculation was performed considering the teeth that were analyzed.

## Results

**Figure 3:**
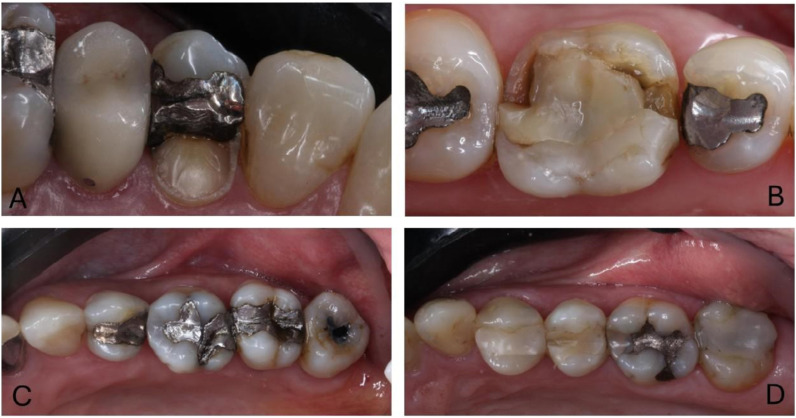
Photographs illustrate the teeth included in the study. Teeth A and B were assigned to the case group, and teeth C and D were assigned to the control group.

Legend: A—fracture with amalgam restoration (L); B—fracture with composite resin (M-O-D); C—amalgam restoration; D—composite resin (M-O-D).

Figure 3 provides representative photographs documenting the cases. The case group consisted of selected fractured teeth with restorations, categorized as either amalgam or composite resin restorations, representing the most common types of restorations observed. The control group cases are also represented.

**Figure 4:**
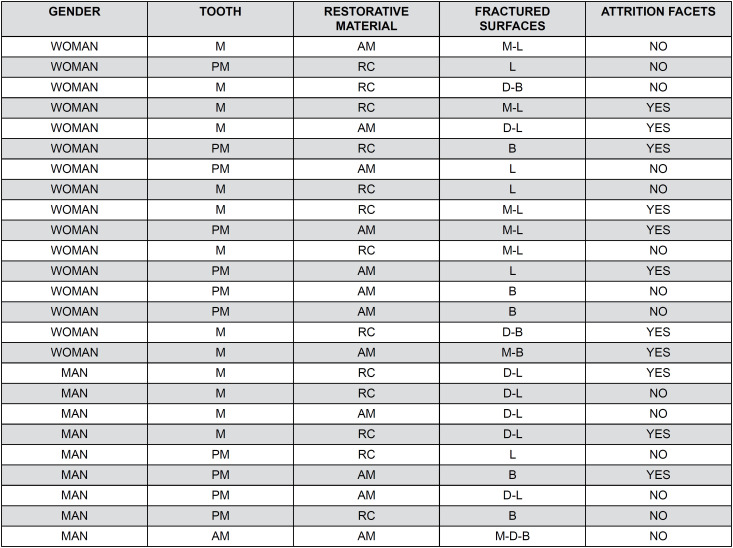
Demographic data of the case group considering both genders.

Legend: M – molar; PM – premolar; RC – Composite resin; AM – Amalgam; L – lingual surface; B – buccal surface; M – mesial surface; D – distal surface.

In the case group, 13 patients had fractured teeth with composite resin restorations, and 12 patients had fractured teeth with amalgam restorations. Among the 25 patients in the case group, 11 exhibited attrition facets. Within this subgroup, fractures with amalgam restorations were observed in five patients and composite resin restorations in six patients—only nine out of the 25 patients presented fractures limited to a single tooth surface (Figure 4).

**Figure 5:**
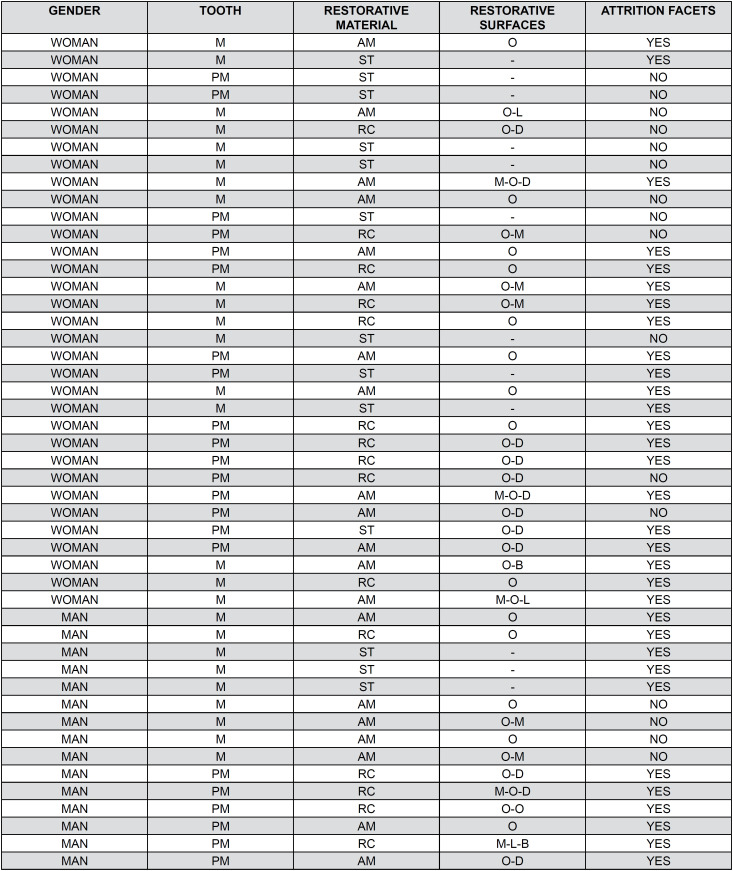
Demographic data of the case group considering both genders.

Legend: M – molar; PM – premolar; RC – Composite resin; AM – Amalgam; ST – Sound tooth; L – lingual surface; B – buccal surface; M – mesial surface; D – distal surface.

The control group was comprised of 50 patients, double the number included in the case group. Among these, 20 patients presented well-maintained amalgam restorations; 13 patients exhibited attritional facets within this subgroup. Fifteen patients had clinically acceptable composite resin restorations, with 12 of these exhibiting wear on the anterior dentition. Additionally, 13 patients with intact teeth were included in the control group, and seven of these presented attrition wear (Figure 5).

Regarding the restoration size in the case group, the mean value and standard deviation are 4.07±1.58, compared to 3.74±1.43 in the control group. For vertical direction fractures (cervical-incisal direction), only measured in the case group, the mean value is 3.65±1.65. In contrast, horizontal fractures (buccal-lingual direction) have a mean value of 2.42±1.39.

Teeth were categorized according to the presence or absence of amalgam restorations for the statistical analysis of the primary outcome. There was no statistical difference (p=0.848) between the presence or absence of amalgam restorations regarding the risk of tooth fracture. The frequency of occurrence of amalgam restorations was 45.8% in the case group, and 41.7% in the control group. The fracture risk was 1.1 times the odds of fracture when amalgam restorations were present. When intact teeth are excluded, the chances of failure are 0.77 for amalgam restorations, with no statistical difference (p=0.4382).

When teeth with restorations larger than 3.5 mm are considered in the case group, 17.6% showed amalgam restorations and 23.5% had composite resin restoration. In the control group, 26.5% of patients had amalgam restorations and 32.4% had composite resin.

The chances of failure are 0.53 for amalgam restorations in that analysis, with no statistical differences (p=0.433). When teeth with restorations smaller than 3.5 mm are included, the chances of failure are 1.015 for amalgam restorations, with no statistical differences (p=0.983). When teeth with wear facets are included, at least 52.9% of women in the case group presented that condition; in the control group more than 70% of participants did not present wear facets. The odds of failure are 1.357 for amalgam restorations, with no statistical differences (p=0.65).

## Discussion

This case-control study investigated the association between dental fractures in posterior teeth and the presence of amalgam or composite resin restorations. The findings revealed no statistically significant association between the type of restorative material and fracture risk. This supports the null hypothesis that there is no association between fractured teeth and the presence of restorative materials. This result aligns with prior research suggesting comparable fracture risks for different restorative materials when factors like restoration size and remaining tooth structure are considered.^16^

The lack of a significant difference in fracture risk aligns with the theory that the restorative material itself may not be the primary determinant of fracture but rather the extent of tooth preparation and remaining tooth structure.^10^ In our study, teeth with restorations larger than 3.5 mm (with at least two surfaces involved) showed a slightly higher failure rate, but the differences were not statistically significant. This highlights the importance of preserving as much natural tooth structure as possible, regardless of the restorative material used.

While secondary outcomes (restoration size and presence of attritional wear) also showed no association with a specific restorative material, it is acknowledged that greater loss of tooth structure during cavity preparation weakens teeth and concentrates occlusal forces.^14^ The present clinical study, which included vital posterior teeth (premolars and molars) with varying restoration sizes and depths, highlights the importance of considering the clinical context, in which forces are applied differently during function and over extended periods compared to *in vitro* compression tests.^15-22^

Another factor that can contribute to tooth fracture is the presence of parafunctional habits or excessive chewing efforts, which can manifest as wear facets.^10^ Patients exhibiting wear facets are believed to exert excessive force during habitual activities and/or functional use. However, in the present study, no statistical differences were observed regarding the presence of wear facets between the two groups. Furthermore, when comparing sound teeth to those restored with amalgam, no statistical differences were found.

Attritional wear was more frequently observed in female participants, particularly in the case group. Specifically, eight out of the 11 case group participants exhibiting attritional wear were female, compared to 21 out of the 33 control group participants with incisal wear. This finding suggests a potential association between parafunctional habits and fracture risk, as attritional wear is indicative of excessive occlusal forces, which can weaken teeth over time.^27^ Gender-specific differences in parafunctional habits, such as bruxism, may partially explain the higher prevalence of attritional wear observed in women. Furthermore, hormonal factors have been proposed to influence bone density and tooth structure, potentially predisposing women to fractures.^16,28^ As a limitation of the study, gender selection was not evenly distributed, as it was not the main objective of the study, a fact that might have underpowered that information. Further studies with that objective might be performed for a better understanding of the gender influence on dental fractures associated with amalgam restorations.

Socioeconomic factors also play a critical role in restorative material selection and subsequent outcomes. Patients with limited access to dental care may delay treatment, leading to larger restorations and increased fracture risk. Additionally, economic constraints may influence the choice of materials, with amalgam often being preferred due to its lower cost despite its declining popularity in recent years.^29^ It is important to highlight that patients receiving treatments at a public university in Brazil usually have limited financial resources, and procedures such as indirect restorations, which mostly involve laboratory costs, are postponed to the maximum. To restore the function of the remaining tooth structure in those scenarios, dentists often extended the indications for direct restorative treatments.

The literature presents numerous studies on direct restorations in posterior teeth; however, few reports have reported dental fractures associated with the type of direct restorative material.^11-13^ Most available studies primarily focus on correlating the characteristics of amalgam and composite resin with potential failures, intending to assess the longevity of restorations in clinical service.^14^ While esthetic requirements are a significant consideration, mechanical properties, longevity, and functional rehabilitation should be the fundamental criteria for selecting a restorative material.^16^ Nevertheless, from a clinical perspective, there is not enough literature that investigates the relationship between restorative material and its influence on dental fractures after restorations in clinical service.

Thus, because this is a case-control study, with patients coming from treatments performed at the university itself (with undergraduate students) and in private practices, the operator factor cannot be controlled, and, thus, analyses regarding the professional experience cannot be performed.

Regarding amalgam, although the restorative technique is clinically less sensitive and easier to perform than the adhesive material, it requires greater dental preparation.^30,31^ Therefore, it is understood that the operator factor is important for the longevity of the restorations^32,33^ and the tooth’.

Several factors may have influenced the present findings. The non-randomized patient selection from a university clinic may not fully represent the general population. Furthermore, the sample size may have been underpowered, and the inclusion criteria for the control group may have led to an imbalance in tooth distribution, with fewer sound teeth in the case group. A key limitation is the inability to determine if resin restorations replaced previous amalgam restorations, potentially confounding the results. The lack of data on restoration lifespan also limits the interpretation of fracture outcomes, as fatigue is a significant factor. More detailed information would be ideal

Despite the limitations presented, the results were positive and reinforced the idea of minimally invasive dentistry. Since there was no statistical difference, it is understood that the exchange of a silver amalgam restoration for an adhesive composite resin restoration is not necessary, unless there is a material failure. It is known that composite resin is the material of choice for posterior restorations because it allows a rehabilitative adhesive protocol without the need to remove sound dental structures for retention.^6^ This recommendation can be extended to cases involving cracks, as the decision to perform restorative treatment on a cracked tooth should be based on a comprehensive set of observations, including symptomatology and the extent of the crack, rather than solely on the presence of the clinical condition, as these are commonly present in teeth restored with silver amalgam.

Considering that only 3% of cracked teeth progress to dental fractures and only 12% show crack progression over three years, the indication for replacing these restorations should not be based solely on the presence of a crack.^34^ Some professionals may opt to replace these restorations when a crack is detected. However, if the restorative material is intact and the patient is asymptomatic, preserving the existing restoration and conducting clinical monitoring align with the principles of minimally invasive prosthodontics.^34^

Thus, embracing the concept of minimal invasion also involves considering and delaying the death cycle of the tooth associated with successive restoration changes. Each time a restoration is replaced, a small portion of the healthy tooth structure is inevitably lost. Future studies should be conducted to enhance our understanding of tooth fractures and all the factors that may contribute to this failure.

## Conclusion

Given the proposed methodology and the limitations of this study, we can conclude that there is no association between tooth fractures and the presence of restorative material. Therefore, the exchange of an amalgam restoration for a composite resin restoration should not be indicated unless there is a failure in the existing restoration.

Data availability

The datasets generated during and/or analyzed during the current study are available from the corresponding author on reasonable request.
